# Prevalence of micronutrient deficiency in popular diet plans

**DOI:** 10.1186/1550-2783-7-24

**Published:** 2010-06-10

**Authors:** Jayson B Calton

**Affiliations:** 1 Department of Nutritional Research and Education, Calton Nutrition, North Venice, FL, USA

## Abstract

**Background:**

Research has shown micronutrient deficiency to be scientifically linked to a higher risk of overweight/obesity and other dangerous and debilitating diseases. With more than two-thirds of the U.S. population overweight or obese, and research showing that one-third are on a diet at any given time, a need existed to determine whether current popular diet plans could protect followers from micronutrient deficiency by providing the minimum levels of 27 micronutrients, as determined by the U.S. Food and Drug Administrations (FDA) Reference Daily Intake (RDI) guidelines.

**Methods:**

Suggested daily menus from four popular diet plans (*Atkins for Life *diet, *The South Beach Diet*, *the DASH diet*, *the DASH diet*) were evaluated. Calorie and micronutrient content of each ingredient, in each meal, were determined by using food composition data from the U.S. Department of Agriculture Nutrient Database for Standard Reference. The results were evaluated for sufficiency and total calories and deficient micronutrients were identified. The diet plans that did not meet 100% sufficiency by RDI guidelines for each of the 27 micronutrients were re-analyzed; (1) to identify a micronutrient sufficient calorie intake for all 27 micronutrients, and (2) to identify a second micronutrient sufficient calorie intake when consistently low or nonexistent micronutrients were removed from the sufficiency requirement.

**Results:**

Analysis determined that each of the four popular diet plans failed to provide minimum RDI sufficiency for all 27 micronutrients analyzed. The four diet plans, on average, were found to be RDI sufficient in (11.75 ± 2.02; mean ± SEM) of the analyzed 27 micronutrients and contain (1748.25 ± 209.57) kcal. Further analysis of the four diets found that an average calorie intake of (27,575 ± 4660.72) would be required to achieve sufficiency in all 27 micronutrients. Six micronutrients (vitamin B7, vitamin D, vitamin E, chromium, iodine and molybdenum) were identified as consistently low or nonexistent in all four diet plans. These six micronutrients were removed from the sufficiency requirement and additional analysis of the four diets was conducted. It was determined that an average calorie content of (3,475 ± 543.81) would be required to reach 100% sufficiency in the remaining 21 micronutrients.

**Conclusion:**

These findings are significant and indicate that an individual following a popular diet plan as suggested, with food alone, has a high likelihood of becoming micronutrient deficient; a state shown to be scientifically linked to an increased risk for many dangerous and debilitating health conditions and diseases.

## Background

Several scientific studies have established a strong correlation between nutrient deficiency and the condition of overweight/obesity, including one study that found an 80.8% increased likelihood of being overweight or obese in micronutrient deficient subjects [1-4]. In addition, sub-optimal intake of certain micronutrients is an established factor in a multitude of dangerous health conditions and diseases, including resistance to infection, birth defects, cancer, cardiovascular disease and osteoporosis [5-7]. According to the latest statistics from the Centers for Disease Control and Prevention (CDC), America's overweight/obesity epidemic now affects more than two out of three adults and 16% of children. Its obese population is now greater than its overweight population with more than 34% of American adults obese. This has caused a sharp increase in the number of dieting attempts undertaken by overweight or obese individuals with the intent to lose weight and/or improve their health. According to a nationwide survey by the Calorie Control Council, there are more than 65 million Americans currently on a diet of some kind, equating to approximately 25% of all adults in America. These facts create a clear need to examine whether the popular diet plans millions of people are following to help them lose weight and/or improve health, can provide at least minimum micronutrient sufficiency, when followed as suggested, with a food only approach. While micronutrient sufficiency research on random diet profiles has been conducted [8] showing high levels of micronutrient deficiencies (40.5%), no studies were found that investigated specific popular diet plans designed to promote weight loss and/or improve health.

This study examined three days of suggested daily menus from each of the four popular diet plans to determine, if when followed as directed, they delivered 100% RDI sufficiency of 27 essential micronutrients. The 27 essential micronutrients used in this study were: vitamin A, vitamin B1 (thiamine), vitamin B2 (riboflavin), vitamin B3 (niacin), vitamin B5 (pantothenic acid), vitamin B6, vitamin B7 (biotin), vitamin B9 (folate), vitamin B12, vitamin C, vitamin D, vitamin E, vitamin K, choline, Ca, (calcium), Cr (chromium), Cu (copper), Fe (iron), I (iodine), K (potassium), Mg (magnesium), Mn (manganese), Mo (molybdenum), Na (sodium), P (phosphorus), Se (selenium), and Zn (zinc). In the case of choline, the established Dietary Reference Intake (DRI) was used because an RDI for choline has not been established. It should also be noted that although Cr (chromium) is included in the RDI and has an established reference level, it is not considered an essential nutrient. Any reference to the like should be disregarded. Each popular diet plan was evaluated separately. Three suggested daily menus were selected for each diet plan. Each ingredient from each selected daily menu was entered into the database and was evaluated for their micronutrient levels and calories. The three daily menus were then averaged and sufficiency for the 27 micronutrients was tested based on the RDI guidelines. If 100% micronutrient sufficiency was not achieved for each of the 27 micronutrients then the calorie level was uniformly increased, according to each plan's unique macronutrient ratio, until nutrient sufficiency was achieved for all 27 micronutrients revealing an RDI micronutrient sufficient calorie intake for each popular diet plan. The study then used the results from these observations to answer four original research questions:

1. At the recommended calorie intake levels for each diet plan, what percentage of the RDI for each of the 27 essential micronutrients is being delivered from whole food alone?

2. What percentage of the diet plans examined, if followed as directed using whole food alone, are micronutrient sufficient based on the RDI for all 27 essential micronutrients?

3. At what calorie intake levels do the suggested menus of the diet plans examined allow an individual to receive minimum RDI sufficiency of all 27 essential micronutrients from whole food alone?

4. Which, if any, of the 27 essential micronutrients are deficient most often within the four popular diet plans examined and does a pattern exist?

## Methods

The conducted study had no human participants. It analyzed the sufficiency level of 27 essential micronutrients within four popular diet plans (the *Atkins For Life *diet, a low-carbohydrate plan, *Atkins For Life*, a Mediterranean style diet plan, *The South Beach Diet*, a medically based plan recommended by a wide variety of medical and governmental organizations, including the Mayo Clinic, to reduce high blood pressure, and *the DASH diet*, a low-fat plan), exactly as they were recommended in their respected texts, official companions, or related web sources, using the U.S. Department of Agriculture Nutrient Database for Standard Reference [9] as the major source of food composition data and the World's Healthiest Foods databases as a secondary source[10]. Each diet was analyzed to determine the daily and three day average of essential micronutrient levels provided compared to the amounts suggested by the U.S. Food and Drug Administrations (FDA) RDI guidelines appropriate for healthy adult men and women between the ages of 18-55, excluding pregnant and lactating women. To determine the three day average, each ingredient in each meal was individually calculated, based on serving size, for calories and its content of 27 essential micronutrients. On average, 15 meals and 75 ingredients were evaluated for each of the four popular diet plans. Depending on the sufficiency level, the calories for each plan were uniformly raised or lowered, as necessary, so that each plan's unique macronutrient ratio remained the same as the original, until 100% RDI sufficiency for each of the 27 essential micronutrients was met. This study also evaluated and recorded a revealed pattern of commonly deficient and/or non-existent micronutrients in each diet plan. Once identified, these deficient and/or non-existent micronutrients were removed from the sufficiency requirements and a re-analysis was then preformed to determine a sufficiency calorie intake for the remaining micronutrients.

## Results

### Sufficiency Analysis

**Figure 1:**
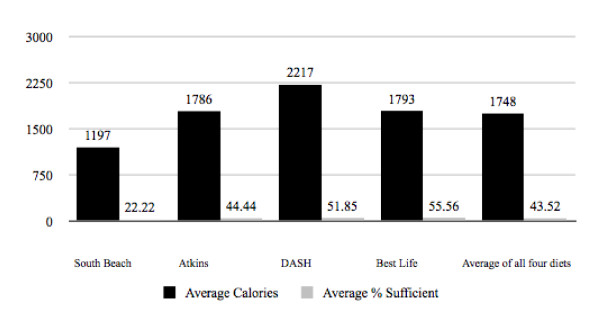
**Average Calorie Intake and Sufficiency Percentages of Suggested Daily Menus**.

It was found that all four diet plans failed to deliver 100% sufficiency for the selected 27 essential micronutrients, based on RDI guidelines, when followed as recommended by their suggested daily menus using whole food alone. Analysis revealed that the *Atkins for Life *diet was (44.44%) sufficient, delivered 100% RDI sufficiency for 12 out of 27 essential micronutrients and contained 1,786 calories. *The Best Life Diet *was (55.56%) sufficient, delivered 100% of the RDI for 15 out of 27 essential micronutrients and contained 1,793 calories. The *DASH *diet was (51.85%) sufficient, delivered 100% of the RDI for 14 out of 27 essential micronutrients and contained 2,217 calories. Lastly, *The South Beach Diet *was (22.22%) sufficient, delivered 100% RDI sufficiency for 6 out of 27 essential micronutrients and contained 1,197 calories. The overall average micronutrient sufficiency percentage and calorie content of all four diets was (43.52%) sufficiency and 1,748 calories. It was found that a typical dieter, using one of these four popular diet plans would be, on average, 56.48% deficient in obtaining RDI sufficiency, leaving them lacking in 15 out of the 27 essential micronutrients analyzed (Figure 1, Table 1).

**Table 1:** Micronutrient Sufficiency Comparisons for Recommended Daily Menus

MICRONUTRIENTS	% Reference Daily Intake (RDI)				
	**SB**	**AFL**	**DASH**	**BL**	**AVERAGE**

VITAMIN A	332%	342%	243%	132%	262%
VITAMIN B1	66%	108%	120%	123%	104%
VITAMIN B2	94%	103%	161%	154%	128%
VITAMIN B3	94%	130%	145%	79%	112%
VITAMIN B5	45%	57%	72%	58%	58%
VITAMIN B6	90%	121%	174%	163%	137%
VITAMIN B7	7%	8%	12%	90%	29%
VITAMIN B9	83%	113%	131%	136%	116%
VITAMIN B12	80%	140%	95%	138%	113%
VITAMIN C	289%	318%	186%	259%	263%
VITAMIN D	51%	70%	58%	47%	57%
VITAMIN E	23%	24%	52%	38%	34%
VITAMIN K	288%	160%	437%	247%	283%
CHOLINE	56%	68%	46%	55%	56%
CALCIUM	81%	65%	148%	133%	107%
CHROMIUM	7%	8%	8%	11%	9%
COPPER	52%	65%	109%	98%	81%
IRON	51%	81%	97%	102%	83%
IODINE	32%	36%	50%	16%	34%
POTASSIUM	57%	64%	94%	77%	73%
MAGNESIUM	55%	69%	142%	120%	97%
MANGANESE	76%	119%	370%	281%	212%
MOLYBDENUM	37%	85%	35%	740%	224%
SODIUM	101%	77%	95%	107%	95%
PHOSPHORUS	127%	135%	223%	180%	166%
SELENIUM	202%	137%	223%	201%	191%
ZINC	57%	98%	95%	85%	84%

					

Total Calories	1197	1786	2217	1793	1748
# of Deficient Micronutrients	21	15	13	12	15
Sufficiency Percentage	22.22%	44.44%	51.85%	56.56%	43.52%

South Beach (SB), Atkins For Life (AFL), DASH diet (DASH), Best Life (BL)

### A Reanalysis for 100% sufficiency

**Figure 2:**
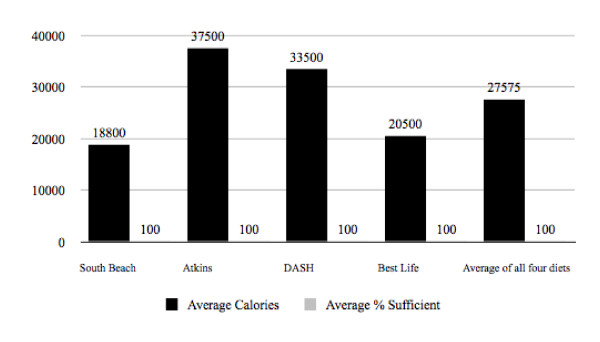
**Average Calorie Intake Required to Reach 100% Sufficiency in 27 Essential Micronutrients**.

In accordance with the study's objectives, calories for each program were raised uniformly until 100% RDI sufficiency was achieved. Food selections and macronutrient ratios were kept exactly the same as was indicated in the suggested daily menus. The required amount of those foods was simply raised uniformly until 100% RDI sufficiency was met for all 27 micronutrients. New calorie intakes were calculated and an evaluation determined that the *Atkins for Life *diet required 37,500 calories to become 100% RDI sufficient in all 27 essential micronutrients. *The Best Life Diet *required 20,500 calories to do the same. The *DASH *diet required 33,500 calories and *The South Beach Diet *required the least, at 18,800 calories. On average, the four diets required 27,575 calories to become 100% sufficient in all 27 essential micronutrients based on RDI guidelines. It was noted that this was well over any calorie intake level in which weight loss and/or health benefits could be achieved (Figure 2, Table 2).

**Table 2:** Micronutrient Sufficiency Comparisons - 100% Sufficiency for 27 Micronutrients

MICRONUTRIENTS	% Reference Daily Intake (RDI)				
	**SB**	**AFL**	**DASH**	**BL**	**AVERAGE**

VITAMIN A	5920%	5354%	4042%	1380%	4174%
VITAMIN B1	1155%	3293%	1839%	1567%	1964%
VITAMIN B2	1446%	2307%	2434%	1738%	1981%
VITAMIN B3	1689%	3061%	2144%	1009%	1976%
VITAMIN B5	719%	1272%	1046%	628%	916%
VITAMIN B6	1499%	3162%	2459%	2272%	2348%
VITAMIN B7	100%	117%	195%	109%	130%
VITAMIN B9	1312%	3101%	2130%	1607%	2038%
VITAMIN B12	1311%	3688%	1298%	1842%	2035%
VITAMIN C	4434%	6826%	2696%	2444%	4100%
VITAMIN D	1062%	2712%	864%	541%	1295%
VITAMIN E	334%	404%	895%	409%	511%
VITAMIN K	5364%	3449%	7125%	2071%	4502%
CHOLINE	797%	1286%	726%	535%	836%
CALCIUM	1246%	1779%	2195%	1401%	1655%
CHROMIUM	113%	100%	100%	129%	111%
COPPER	763%	1453%	1748%	1309%	1318%
IRON	743%	1806%	1528%	1270%	1337%
IODINE	416%	425%	656%	100%	399%
POTASSIUM	896%	1389%	1400%	943%	1157%
MAGNESIUM	871%	1723%	2173%	1585%	1588%
MANGANESE	1174%	3601%	5778%	3656%	3552%
MOLYBDENUM	532%	1084%	541%	638%	699%
SODIUM	1582%	1350%	1501%	1139%	1393%
PHOSPHORUS	2015%	3345%	3298%	2216%	2719%
SELENIUM	3121%	3399%	2976%	2295%	2948%
ZINC	841%	2381%	1394%	1069%	1421%

					

Total Calories	18800	37500	33500	20500	27575
# of Deficient Micronutrients	0	0	0	0	0
Sufficiency Percentage	100%	100%	100%	100%	100%

South Beach (SB), Atkins For Life (AFL), DASH diet (DASH), Best Life (BL)

### Determining a pattern

In keeping with the study's objectives, the micronutrients were examined to determine if a pattern of consistently low or non-existent micronutrients existed within the four diet plans. Upon examination, a pattern did reveal itself; six micronutrients were consistently deficient or non-existent within all four diet plans. These micronutrients were vitamin B7 (biotin), vitamin D, vitamin E, Cr (chromium), I (iodine), and Mo (molybdenum).

### An Analysis Based on 21 micronutrients

**Figure 3:**
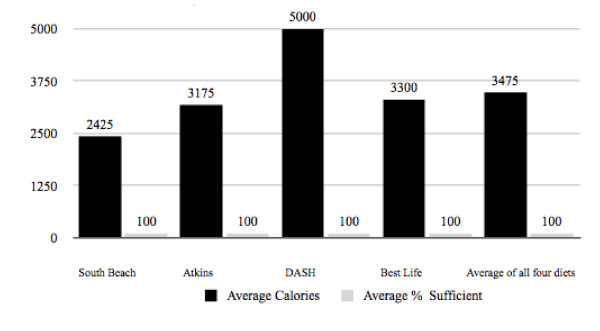
**Average Calorie Intake Required to Reach 100% Sufficiency in 21 Essential Micronutrients**.

Based on the observation it was decided to re-evaluate research question 3 using the remaining 21 micronutrients to see if calorie intake requirement to achieve 100% RDI sufficiency for the remaining 21 micronutrients would become more realistic. It was found that indeed the calorie intake requirement dropped dramatically to 3,475 calories (Figure 3, Table 3). It was noted that this calorie intake was still nearly double the 1,754 calories, which was the average calorie intake of the selected plans when followed using suggested daily menus intended for weight loss and/or health improvement. However, this re-evaluation did suggest that minimum sufficiency for the analyzed 27 essential micronutrients could be achieved at a realistic, medically sound caloric intake with proper micronutrient supplementation.

**Table 3:** Micronutrient Sufficiency Comparisons - 100% Sufficiency for 21 Micronutrients


**MICRONUTRIENTS**	**% Reference Daily Intake (RDI)**				

	**SB**	**AFL**	**DASH**	**BL**	**AVERAGE**

VITAMIN A	688%	604%	503%	240%	509%
VITAMIN B1	141%	190%	270%	230%	208%
VITAMIN B2	188%	182%	365%	282%	254%
VITAMIN B3	203%	229%	332%	148%	228%
VITAMIN B5	92%	100%	167%	106%	116%
VITAMIN B6	187%	212%	411%	309%	280%
VITAMIN B7 ***	14%	14%	26%	17%	18%
VITAMIN B9	168%	199%	285%	250%	226%
VITAMIN B12	166%	248%	234%	260%	227%
VITAMIN C	598%	560%	459%	458%	519%
VITAMIN D ***	118%	124%	135%	87%	116%
VITAMIN E ***	45%	43%	102%	69%	65%
VITAMIN K	631%	284%	982%	434%	583%
CHOLINE	109%	122%	100%	100%	108%
CALCIUM	162%	115%	340%	242%	215%
CHROMIUM ***	15%	15%	20%	20%	18%
COPPER	102%	114%	232%	185%	158%
IRON	100%	144%	211%	191%	162%
IODINE ***	62%	65%	133%	27%	72%
POTASSIUM	115%	113%	216%	143%	147%
MAGNESIUM	110%	122%	316%	226%	194%
MANGANESE	151%	212%	803%	527%	423%
MOLYBDENUM ***	72%	148%	80%	133%	108%
SODIUM	194%	137%	205%	195%	183%
PHOSPHORUS	255%	239%	506%	336%	334%
SELENIUM	404%	243%	553%	370%	393%
ZINC	114%	173%	218%	159%	166%

					

Total Calories	2425	3175	5000	3300	3475
# of Deficient Micronutrients	0	0	0	0	0
Sufficiency Percentage	100%	100%	100%	100%	100%

South Beach (SB), Atkins For Life (AFL), DASH diet (DASH), Best Life (BL)					

*** Indicates Micronutrient has been Removed from Sufficiency Requirement

## Discussion

The debate over whether an individual can obtain RDI sufficiency of 27 essential micronutrients from a balanced, whole food diet alone was answered in this study, as it relates to the four selected popular diet plans. These selected diet plans are presented to the public as sound, healthy, balanced diets. They recommend their followers eat a variety of fresh fruits and vegetables, whole grains and lean proteins, and yet, not a single plan was able to provide RDI sufficiency of the studies selected 27 essential micronutrients at the calorie intake level suggested by their respective sample menus. It was found that 100% sufficiency was possible for all 27 essential micronutrients only when daily calorie intake requirement averaged 27,575 calories. This extreme calorie intake requirement is, in the opinion of this researcher, impossible and/or medically unwise to obtain and/or sustain. Therefore, it is the conclusion of this study that anyone following one of the selected four diet plans using whole food alone would be, on average, deficient in 56%, or 15 out of 27 essential micronutrients analyzed in this study based on RDI guidelines.

The implications of this study are significant and far-reaching. Micronutrient deficiency has been shown to cause an 80.8% increase in the likelihood of becoming overweight or obese [1] and is scientifically linked to a higher risk of other dangerous and debilitating diseases, including resistance to infection, birth defects, cancer, cardiovascular disease and osteoporosis [5-7]. Consequently, with global obesity being a very real and serious condition it should be of some concern to the millions of individuals worldwide, following one of this study's four popular diet plans, or similar, using whole food alone, that based on the findings of this study, micronutrient deficiency is inevitable. This concern is compounded further by the fact that of the four popular diet plans analyzed, only two, the *Atkins For Life *diet and *The Best Life Diet*, adamantly recommended their followers to take a daily multivitamin.

When questions such as: "Is it true that I can get all the vitamins/minerals I need from the food that I eat?" are answered by the nutritional professionals at nutrition.gov by stating, "It is true that healthy individuals can get all of the vitamins and minerals they need from a well balanced diet," it confuses the general public. It completely disregards the findings of Drs. Fairfield and Fletcher of Harvard University and writers of the new guidelines for the *Journal of American Medical Association (JAMA)*. Dr. Fletcher states, "Even people who eat five daily servings of fruits and vegetables may not get enough of certain vitamins for optimum health. Most people, for instance, cannot get the healthiest levels of folate and vitamins D and E from recommended diets." According to Dr. Fletcher and this study, micronutrient deficiency may be more widespread than commonly thought and may be at the root of the August 31, 2002 urgings of the American Medical Association when it reversed their long-standing anti-vitamin policy by stating, "The Journal of the American Medical Association today is advising all adults to take at least one multivitamin pill each day."

## Conclusions

This study shows a significant prevalence of micronutrient deficiency in popular diet plans. It is the conclusion of this researcher that an individual following a popular diet plan using food alone, has a high likelihood of becoming micronutrient deficient, a condition shown to be scientifically linked to a higher risk of dangerous and debilitating diseases including cancer, osteoporosis, heart disease, birth defects and overweight/obesity. Based on this study's findings, the belief that a healthy, balanced diet can consistently deliver, to a typical dieter, all of the essential vitamins and minerals they need, through whole food alone, is in dire need of revision. It would appear that supplementation should be considered as a viable, low cost method to achieve micronutrient sufficiency and reduce the risk for some of today's most prevalent and devastating health conditions and diseases. In conclusion, this study recommends that all individuals, particularly those following a popular diet plan, would benefit from and should take a daily multivitamin supplement to fill the nutritional gap between where their whole food diet leaves off and micronutrient sufficiency is achieved.

## Competing interests

JBC is the CEO of Calton Nutrition, a private corporation that researches the causation and prevalence of micronutrient deficiency worldwide. Due to the results of its research Calton Nutrition is in the process of developing a multivitamin.

## References

[B1] Asfaw A Micronutrient deficiency and the prevalence of mothers' overweight/obesity in Egypt Economics and Human Biology 2007 5 471 483 10.1016/j.ehb.2007.03.004 17449338

[B2] Smotkin-Tangorra M Purushothaman R Gupta A Nejati G Anhalt H Ten S Prevalence of vitamin D insufficiency in obese children and adolescents Journal of Pediatric Endocrinology & Metabolism 2007 20 817 823 http://www.ncbi.nlm.nih.gov/pubmed/17849744 10.1515/jpem.2007.20.7.817 17849744

[B3] Dzieniszewski J Jorosz M Szczygie B Diugosz J Marlicz K Linke K Lachowicz A Ryko-Skiba M Orzeszko M Nutritional status of patient hospitalized in Poland European Journal of Clinical Nutrition 2005 59 552 560 10.1038/sj.ejcn.1602117 15714213

[B4] Koleva M Kadiiska A Markovska V Nacheva A Boev M Nutrition nutritional behavior, and obesity Central European Journal of Public Health 2000 8 10 13 10761619

[B5] Fletcher R Fairfield K Vitamins for Chronic Disease Prevention in Adults The Journal of the American Medical Association 2002 287 3127 3129 10.1001/jama.287.23.3127 12069676

[B6] Field C Johnson I Schley P Nutrients and their role on host resistance to infection Journal of Leukocyte Biology 2002 71 16 32 11781377

[B7] Combs G Jr Status of selenium in prostate cancer prevention British Journal of Cancer 2004 91 195 199 15213714 10.1038/sj.bjc.6601974 PMC2409825

[B8] Misner B Food alone may not provide sufficient micronutrients for preventing deficiency Journal of the International Society of Sports Nutrition 2006 3 51 55 10.1186/1550-2783-3-1-51 18500963 PMC2129155

[B9] USDA national nutrient database for standard reference(Release 20) http://www.ars.usda.gov/ba/bhnrc/ndl

[B10] World's Healthiest Foods Database Food Processor for Windows nutrition analysis software, version 7.60. Salem/ESHA Research, PMID: 17800 http://www.whfoods.com

